# Cognitive Impairment among Patients with Chronic Obstructive Pulmonary Disease Compared to Normal Individuals

**Published:** 2017

**Authors:** Mitra Samareh Fekri, Seyed-Mehdi Hashemi-Bajgani, Ahmad Naghibzadeh-Tahami, Fateme Arabnejad

**Affiliations:** 1 Cardiovascular Research Center, Institute of Basic and Clinical Physiology Sciences, Kerman University of Medical Sciences, Kerman, Iran; 2 Department of Internal Medicine, Kerman University of Medical Sciences, Kerman, Iran; 3 Social Determinants of Health Research Center, Institute for Futures Studies in Health, Kerman University of Medical Sciences, Kerman, Iran; 4 Kerman Medical Students Research Center, Kerman University of Medical Science, Kerman, Iran.

**Keywords:** COPD, Cognitive impairment, MMSE questionnaire, Smoking

## Abstract

**Background:**

Chronic obstructive pulmonary disease (COPD) is one of the most important causes of morbidity and mortality worldwide. The complications of COPD are numerous, and cognitive impairment is one of the most common complications that relates to mortality and morbidity directly. The present study was conducted with the aim of evaluating the prevalence of cognitive impairment in patients with COPD in comparison to normal individuals.

**Materials and Methods:**

In this case-control study, 87 patients with COPD, whose diagnoses were confirmed by a pulmonologist based on the spirometry test findings, were included. The mini-mental state examination (MMSE) questionnaire was administered for assessing the cognitive impairment. Arterial oxygen saturation was measured. The MMSE questionnaires were administered to 60 healthy, age-and-sex-matched individuals without a history of myocardial infarction or cerebrovascular infarction, and their arterial oxygen saturations were measured. The data were analyzed using the SPSS (version 20) software.

**Results:**

In the case group, 42 patients (48.27%) had no cognitive impairment, 39 (44.82%) had mild, and 6 (6.89%) had moderate cognitive impairment. In the control group, 38 (63.33%) had no cognitive impairment, 20 (33.33%) mild and 2 (3.33 %) moderate cognitive impairment. There were significant relationships between the cognitive impairment and arterial oxygen saturation, severity of COPD, and higher age. The prevalence of cognitive impairment was 51.71% in the case group and 36.66% in the control group.

**Conclusion:**

According the results of the present study, COPD increased the risk of cognitive impairment significantly and is related to the severity of COPD, arterial oxygen saturation, and higher age.

## INTRODUCTION

Chronic obstructive pulmonary disease (COPD) is characterized by an irreversible limitation of pulmonary air flow and a decrease in the FEV1/FVC proportion. It is a main cause of mortality and morbidity in all countries. The most common age of onset is more than 55 years. More than 14% of individuals aged more than 65 years have COPD; an increase in its prevalence and mortality is predicted in coming decades(1). The traditional criteria for assessing the severity of COPD, such as restriction of air flow, cannot clearly define the prognosis; this is mainly because it does not consider the multi-system nature of this disease. Some of the complications of COPD are cardiovascular and skeletal complications, hypertension, metabolic disorders, depression, and cognitive disorders. Cognitive impairment is the most common extra-pulmonary manifestation of COPD and is related to the mortality and disability of these patients; however, the mechanism for the same is not well understood. A systematic review reported that the cognitive function of patients with COPD is impaired in relation to normal people(2). COPD is a major risk factor for cognitive disorders(3). The prevalence of cognitive disorders in these patients has been reported to be 10% to 48%(4), and COPD can increase the risk of cognitive disorders by approximately 2.5 times(5–7). There is a direct relationship between the severity of COPD and cognitive disorders (2, 8–10). However, some studies could not find any association between COPD and cognitive disorders(1). Concurrency of COPD and cognitive disorders leads to an increase in the mortality and hospitalization due to all causes and not pulmonary causes alone(11). In a systematic review, cognitive disorders were found in the severe form of COPD alone(12). It is known that brain hypo-perfusion occurs in patients with COPD and that an important cause for cognitive impairment is the lack of oxygen usage in hypoxemic patients(5). However, cognitive disorders have been found even in non-hypoxemic patients in some studies(13).

To have a better understanding of COPD, we need to know its complications better, including cognitive impairment. Considering the high prevalence of COPD, concurrence of cognitive impairment in these patients, and the discrepant results of different studies, we decided to assess the cognitive impairment in patients with COPD and the relation of severity of cognitive impairment using some variables, such as age, sex, arterial oxygen saturation, and use of oxygen at home.

## MATERIALS AND METHODS

In this case-control study, with the ethics code k/93/178 from the Kerman Medical University Ethics Committee, 87 patients with a history and symptoms of COPD were assessed by a pulmonologist. These patients underwent the spirometry test using a Spirolab3 (MIR) spirometer made in Italy. The goals of the study were described to the patients, and the informed consent was obtained from each patient. Patients were considered to have COPD if they had FEV1/FVC < 0.7, and if it remained < 0.7 fifteen minutes after the administration of two puffs of the salbutamol inhaler; moreover, their FEV1 should not have increased by 12% or 200 cc. Patients with COPD were divided into mild, moderate, severe, and very severe, according to the Global Initiative for Chronic Obstructive Lung Disease (GOLD) criteria.

The exclusion criteria were illiteracy and history of myocardial infarction or cerebrovascular accident. The MMSE questionnaire was administered to all the patients by the interviewer. The MMSE questionnaire had 11 questions and 30 points that assessed cognitive disorders involving registration, orientation, recent memory, attention, calculation, spatial thinking, and verbal function domains. The arterial oxygen saturation was measured using a pulse oxymeter (Zyklusmed, Germany) and questions regarding the age, sex, job, number of COPD exacerbations during the past year, and systemic diseases, were filled. An arterial oxygen saturation below 90% was considered hypoxemia. A total of 60 healthy, age-and-sex-matched people without a history of CVA or MI were referred to the pulmonologist with pulmonary complaints; COPD was ruled out by history-taking and spirometry; these individuals were considered as the control group. The MMSE questionnaire was also administered.

All cases and controls were divided into three groups for cognitive impairment, according to the MMSE score: mild (19 ≤ score < 23), moderate (10 ≤ score < 19) and severe (score<10). T-test was done to compare the MMSE scores between the two groups and the regression test was performed to assess the relation of the above-mentioned variables with the MMSE score.

## RESULTS

In the case group of 87 patients, 8 (9.2%) were women and 79 (90.8%) were men; in the control group, 19 (31.67%) were women and 41 (68.33%) were men. The average age of the cases was 60.47 ± 9.83 years (range, 40–83 years) and that of the controls was 58.15 ± 9.8 years (range, 40–80 years). In patients with COPD, 7 (8.04%) had mild, 35 (40.23%) moderate, 29 (33.33%) severe, and 16 (18.39%) very severe forms of COPD. In the case group, 7 (8%) patients had a history of baking bread in traditional furnaces and 16 (18%) had jobs that were high-risk for pulmonary diseases, such as working in mines or cement factories. In the case and control groups, 12.6% and 36.66% had no history of cigar or opium consumption, respectively. When the regression test was performed for each of the variables separately, there were significant relationships between the MMSE score and arterial oxygen saturation, age, FEV1%, number of coexisting systemic diseases (diabetes, chronic kidney disease, and hypertension), education, number of exacerbations, and amount of cigar and opium consumption. However, there were no significant relationships between the MMSE score and sex, high-risk job, and history of baking. When the variables that had a significant relationship with the MMSE score were entered into the final model for linear regression analysis, there was a significant relationship between the MMSE score and just three variables: age, O_2_ saturation, and FEV1% (Table 1).

**Table 1.** The relationship between MMSE score and age, O2 saturation and %FEV1

**Variable**	**B coefficient**	**p-value**
Age	−0.053	0.019
O2 sat	0.124	0.018
FEV1	0.21	0.042

**Figure 1.**
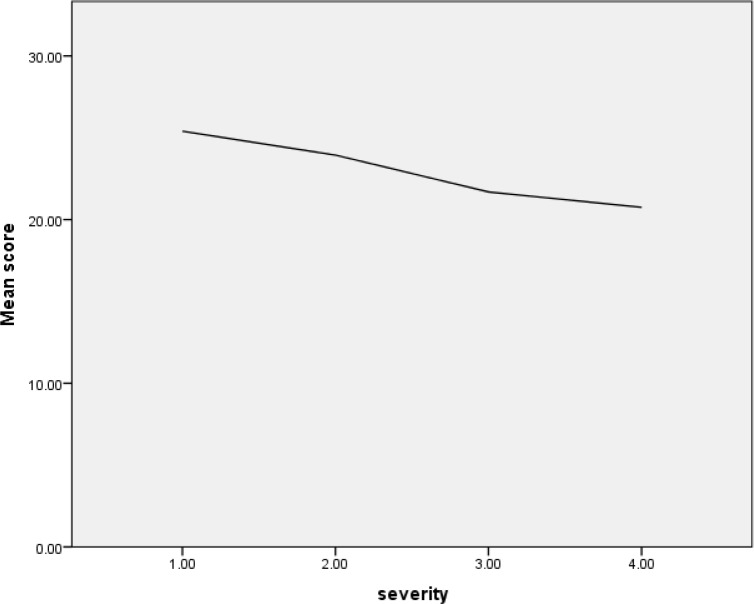
Score of MMSE questionnaire according to severity of COPD in the case group.

In the case group, 42 (48.27%) patients had no cognitive impairment, 39 (44.82%) had mild and 6 (6.89%) had moderate cognitive impairments. In the control group, 38 (63.33%) patients had no cognitive impairment, 20 (33.33%) had mild and 2 (3.33%) had moderate cognitive impairments. There was a significant relationship between FEV1% and the MMSE score (p-value < 0.0001) and an inverse relationship between the severity of COPD and MMSE score (p-value < 0.0001; Figure 1).

There was a significant difference between the MMSE scores of the cases and controls (p-value < 0.0001; mean difference, 1.12). The MMSE scores were 23.63 ± 2.8 in the control group and 22.51 ± 2.4 in the case group.

There was a significant difference between the MMSE scores of the control group and the scores of non-hypoxemic patients with COPD in the case group (p-value < 0.001; mean difference, 0.7).

To answer the question of which domains of cognition were more impaired due to the severity of COPD, bivariate and Kendall correlation analyses were performed; according to them, the relationship of the severity of COPD was significant with questions 1, 4, 5, 7, and 11; these questions were related to the time orientation, calculation, recent memory, attention, and spatial thinking, respectively (Table 2).

**Table 2.** The relationship between %FEV1 and the questions of the MMSE questionnaire

**No. of question**	**Correlation Coefficient**	**p-value**
1	0.13	0.042
2	−0.057	0.398
3	−0.079	0.247
4	0.432	0.000
5	0.489	0.000
6	-	-
7	0.377	0.000
8	0.031	0.654
9	-	-
10	0.081	0.238
11	0.209	0.002

## DISCUSSION

In the present study 51.71% of patients with COPD and 36.66% of the control group had cognitive impairment; these values were 36% and 12%, respectively in a study that was conducted in 2012(2). In some studies, cognitive impairment was found in severe COPD alone (7). In the previous study that had a 20-year follow up for patients with COPD, it was shown that COPD led to a two-fold increased risk of cognitive disorders and Alzheimer disease (14). The prevalence of cognitive disorders among patients with COPD varies from 61% to 27% in different studies, according to the patient selection and severity of the disease (12, 15). However, the difference in cognitive impairment between the case and control groups was not significant in some studies (10, 16); this differed from the findings of our study.

Short-term hypoxemia and short-term oxygen therapy have no effect on cognitive impairment. In a study conducted on patients with COPD who experienced short-term hypoxemia because of air travel, there was no significant change in the cognitive impairment(17). In another study, there was no change in the cognitive impairment and no improvement in driving after the use of oxygen therapy during driving in patients with COPD(18).

In a study conducted in 2010, the role of hypoxemia and home oxygen therapy in cognitive impairment was well-defined(4). A systematic review conducted in 2012 reported that there was a direct relationship between the severity of cognitive impairment, and hypoxemia and the severity of COPD; however, the effect of this cognitive impairment on the quality of life and daily activity of the patient is not understood to date(19).

In a study on hypoxemic patients with COPD, it was shown that three months of oxygen therapy led to an improvement in the cognition, blood flow in the brain, and autonomic nervous system function; however, the amount of improvement was not statistically significant for any of them(20).

In our study, a low arterial oxygen saturation was one of the most important risk factors for cognitive impairment; however, as the number of patients who used oxygen at home was low (4 of 87 patients), it was not possible to assess the relationship of oxygen therapy with cognitive impairment using statistical analysis. In a study that used magnetic resonance imaging for non-hypoxemic patients with COPD, the white matter integrity and activity of gray matter were reduced in comparison to the control group(8).

In two other studies, it was shown that even nonhypoxemic patients had significant cognitive impairment in comparison to normal people; however, this cognitive impairment did not have a considerable effect on their quality of life(9, 13). In our study, there was a significant difference in the cognitive impairment between nonhypoxemic patients with COPD and the control group.

Another study reported a decrease in the cerebral blood flow of patients with COPD, especially in the frontal area, and patients had a remarkable decrease in the verbal memory, recent memory, and attention, compared to the control group. The decrease in verbal memory was found in all patients with COPD; however, the recent memory and attention impairment were only found in hypoxemic patients(10).

In the study by Antonelli-Incalzi et al., it was shown that the anterior cerebral blood flow was reduced in patients with COPD; moreover, they had impairments in five domains, including spatial thinking, copying a painting, verbal fluency, and recent verbal and visual memory; this was relatively similar to the findings of our study that the recent memory, copying a painting, calculation, and attention were impaired, compared to the control group(21).

We could have assessed the role of home oxygen therapy on the cognitive impairment of patients with COPD if we had more patients who used oxygen at home. If the quality of life in patients with COPD was assessed, we could have understood the clinical effects of this cognitive impairment in the quality of life these patients.

## CONCLUSION

In the present study, there was a significant difference in the MMSE scores between the case and control groups; patients with COPD had a higher risk for cognitive impairment, compared to the control group. The prevalence and severity of cognitive impairment was increased in patients with COPD, compared to the control group; moreover, there was a significant inverse relationship between the severity of COPD and the MMSE score. There was a significant relationship between the MMSE scores and the arterial oxygen saturation, age, and FEV1%. Regarding the questions of the MMSE questionnaire, patients with COPD had impairments in time orientation, recent memory, attention, and spatial thinking. There was a significant difference between the MMSE scores in non-hypoxemic patients with COPD and those in the control group; this indicates that although arterial oxygen saturation is an important factor for cognitive impairment, not all cases of worsening of cognitive impairment in patients with COPD can be attributed to hypoxemia.
